# *Scutellaria baicalensis* Attenuated Neurological Impairment by Regulating Programmed Cell Death Pathway in Ischemic Stroke Mice

**DOI:** 10.3390/cells12172133

**Published:** 2023-08-23

**Authors:** Ho-won Seo, Tae-Young Ha, Geon Ko, Aram Jang, Ji-Woong Choi, Dong-hun Lee, Keun-A Chang

**Affiliations:** 1 Department of Health Science and Technology, Gachon Advanced Institute for Health Sciences & Technology, Gachon University, Incheon 21999, Republic of Korea; tjghdnjs45@gachon.ac.kr (H.-w.S.); sirius9725@gachon.ac.kr (G.K.); 2 Neuroscience Research Institute, Gachon University, Incheon 21565, Republic of Korea; heroine78@gachon.ac.kr; 3 Department of Herbal Pharmacology, College of Korean Medicine, Gachon University, 1342 Seongnamdae-ro, Sujeong-gu, Seongnam-si 13120, Gyeonggi-do, Republic of Korea; ram0626@gachon.ac.kr; 4 College of Pharmacy and Gachon Institute of Pharmaceutical Sciences, Gachon University, Incheon 21936, Republic of Korea; pharmchoi@gachon.ac.kr; 5 Department of Pharmacology, College of Medicine, Gachon University, Incheon 21999, Republic of Korea

**Keywords:** stroke, *Scutellaria baicalensis*, transient MCAO, photothrombotic stroke model, necroptosis, pyroptosis, cell death

## Abstract

Stroke is a major global health problem that causes significant mortality and long-term disability. Post-stroke neurological impairment is a complication that is often underestimated with the risk of persistent neurological deficits. Although traditional Chinese medicines have a long history of being used for stroke, their scientific efficacy remains unclear. *Scutellaria baicalensis*, an herbal component known for its anti-inflammatory and antioxidant properties, has traditionally been used to treat brain disorders. This study investigated the therapeutic effects of the *Scutellaria baicalensis* extraction (SB) during the acute stage of ischemic stroke using photothrombotic (PTB)-induced and transient middle cerebral artery occlusion (tMCAO) model mice. We found that SB mitigated ischemic brain injury, as evidenced by a significant reduction in the modified neurological severity score in the acute stage of PTB and both the acute and chronic stages of tMCAO. Furthermore, we elucidated the regulatory role of SB in the necroptosis and pyroptosis pathways during the acute stage of stroke, underscoring its protective effects. Behavioral assessments demonstrated the effectiveness of SB in ameliorating motor dysfunction and cognitive impairment compared to the group receiving the vehicle. Our findings highlight the potential of SB as a promising therapeutic candidate for stroke. SB was found to help modulate the programmed cell death pathways, promote neuroprotection, and facilitate functional recovery.

## 1. Introduction

Stroke is a life-threatening condition that poses a significant global health burden and is associated with high disability rates. Stroke can lead to severe brain damage, resulting in brain atrophy and subsequent dysfunction. The consequences of a stroke can be debilitating and may even lead to death in severe cases. Previous research on stroke has primarily focused on neuroprotection, aiming to reduce the extent of tissue damage (infarct region) and exploring thrombolytic strategies. However, current research efforts have expanded to encompass various strategies, such as disrupting the blood–brain barrier (BBB), understanding the mechanisms of apoptosis, and addressing secondary inflammatory reactions following stroke [1].

Necroptosis revealed that the programmed cell death necrosis mechanism [2] is induced in ischemic stroke, triggered by the phosphorylation of receptor-interacting protein kinase-1 (RIPK1), receptor-interacting protein kinase 3 (RIPK3), and mixed lineage kinase domain-like (MLKL) [3]. Multiple studies have demonstrated that ischemic stroke leads to the increased expression and activation of the NLRP3 inflammasome in neurons and glial cells, thereby contributing to brain inflammation [4,5]. The NLRP3 inflammasome complex comprises the NLRP3 protein, ASC adapter proteins, and pro-caspase-1 protein [6]. Activation of the NLRP3 inflammasome triggers the cleavage of gasdermin D (GSDMD), which promotes pyroptosis [7,8,9] and amplifies inflammatory responses [10,11]. Understanding these molecular mechanisms of cell death and connected inflammation in stroke pathology provides valuable insights for the development of novel therapeutic interventions targeting necroptosis, pyroptosis, and the NLRP3 inflammasome pathway. Such interventions have the potential to attenuate tissue damage, reduce inflammation, and improve stroke outcomes.

Herbal medicines have been studied for their potential therapeutic effects in neuroprotection and neurological recovery after ischemic stroke [12,13]. Among these herbal medicines, the *Scutellaria baicalensis* extraction (SB) is known for its ability to inhibit NLRP3 inflammasome activation [14], reduce the generation of reactive oxygen species (ROSs) [15], and mitigate neuronal apoptosis [16] in the context of ischemic stroke [17].

Despite its traditional usage, the scientific understanding of SB’s efficacy in stroke treatment has remained limited. Thus, scientific investigations are crucial to evaluate the effectiveness of SB and elucidate the underlying mechanisms involved. 

Our research aimed at oral administration used in traditional medicine to enhance the understanding of the potential therapeutic benefits of SB in the management of ischemic stroke utilizing two mouse models of ischemic stroke (PTB and tMCAO), although intraperitoneal injection had an advantage in bioavailability compared to oral administration. We investigated the effects of SB on infarct volume and neurological deficits at the acute stage of stroke and examined the role of SB in regulating programmed cell death pathways, specifically necroptosis and pyroptosis. Furthermore, we evaluated long-term recovery outcomes by assessing motor function and cognitive impairment through behavioral assessments following SB administration during the acute phase.

## 2. Materials and Methods

### 2.1. Preparation of Scutellaria baicalensis Extraction

The root of *Scutellaria baicalensis* Georgi was bought from Yaksudang Pharmaceutical Co., Ltd. (Seoul, Republic of Korea). Professor Donghun Lee of the Department of Herbal Pharmacology, College of Korean Medicine, Gachon University, was given a voucher specimen (2009150001). The dried root of *Scutellaria baicalensis* was extracted using a reflux device for 3 h at 85 °C and a 10:1 ratio of 30% ethanol. The extract was purified and concentrated under reduced pressure. Subsequently, the powder was prepared via spray drying, and 43.4% of the extract was obtained. The extract was then freeze-dried at −80 °C. Chromatographic analysis of SB was performed by high-performance liquid chromatography (HPLC) using an 1100 series HPLC system (Agilent, Santa Clara, CA, USA). A Zorbax Eclipse XDB C18 column (4.6 × 250 mm, 5 µm; Agilent) was used for chromatic separation at 30 °C. A 1 mg sample was diluted with 10 mL of 50% methanol and then sonicated for 10 min. The samples were filtered using a 0.45 μm syringe filter (Waters Corp., Milford, MA, USA). The mobile phase components were 0.1% formic acid and acetonitrile, which were applied as follows: 0–3 min, 20%; 3–15 min, 20–45%; 15–20 min, 45–60%; and 20–22 min, 60% Solvent B. A 10 μL injection volume was used to mark the runoff at 276 nm. The analysis was performed in triplicate. *Scutellaria baicalensis* contains more than 100 components, and baicalein is most-promising and well-known component [18]. HPLC-UV was conducted for standardized for baicalein for SB (Figure S1). The SB was standardized to include 0.37 mg/g of baicalein.

### 2.2. Animals

All animal experiments were conducted in compliance with the Animal Care and Use Guidelines of Gachon University, Seoul, Republic of Korea (LCDI-2021-0164, GUICUC-R2020029). Six-week-old male ICR mice (32 ± 2 g) were obtained from DBL Co., Ltd. (Eumseong-gun, Chungbuk, Republic of Korea). The mice were acclimatized to the laboratory conditions for one week before MCAO modeling. The mice were kept in a room on a 12 h light and dark cycle with a constant temperature (22 ± 2 °C) and humidity (50 ± 10%) and provided water and food ad libitum during the acclimatization period. All animal experiments were approved by the Institutional Animal Care and Use Committee of the Lee Gil Ya Cancer and Diabetes Institute of Gachon University.

#### 2.2.1. Photothrombotic Model

Permanent focal cerebral ischemia was caused by PTB of the cortical microvasculature in the male ICR mice, as previously described [19], with some modifications. In short, 2% isoflurane in N_2_O/O_2_ (70:30) was used to anesthetize the mice. After Rose Bengal solution was administered intraperitoneally for 5 min, the body temperature of the mice was measured using a rectal thermometer. Because an ischemic stroke can be influenced by hypothermia, mice were maintained at 37 ± 0.5 °C using a regulated heating system. The heads of the mice were fixed on a stereotaxic apparatus (Jeung Do BIO & PLANT Co., Ltd., Nowon-gu, Seoul, Republic of Korea), and the midline of the scalp was incised using a surgical blade (Paragon, No. 10). After the periosteum was removed with a swab, the bregma was identified and marked with a surgical marker, and the scalp was pinched using mosquito forceps (Jeung Do, H-1304). To prevent light dispersion, which may lead to variability, a cold-light aperture (Carl Zeiss, CL6000 LED, Jena, Germany) was placed 2 mm laterally, as close as possible to the skull. After 5 min, the skull was irradiated with an illuminator for 15 min (if the light device was new, the light intensity was 60). A heating pad was used to maintain a rectal temperature of 37 ± 0.5 °C during the operation. Subsequently, a drop of 0.9% NaCl was added to the skull. The incised skin was sutured using a stapler (Jeung Do, MD 20877/203-1000). The body temperature was then measured and was rechecked after 20 min.

#### 2.2.2. Transient Middle Cerebral Artery Occlusion Model

Ischemic stroke was induced by tMCAO surgery, as previously described [20,21]. The eight-week-old male ICR mice were anesthetized with isoflurane (3% induction; 1.5% maintenance with medical O_2_). Blood flow in the MCA was measured with a laser Doppler flowmeter probe (PERIMED, PeriFlux 6000 system, Järfälla, Sweden) attached to the skull of the MCA region (2 mm posterior to the bregma and 5–6 mm laterally) (Figure S2). The common cerebral artery (CCA) and the external cerebral artery (ECA) were isolated, and then, the ECA and CCA were blocked with a clip (Fine Science Tool, FST18055-04, Pyeong-taek, Republic of Korea). A 20 mm-length 6-0 monofilament (Doccol Corp., Sharon, MA, USA) was inserted through the ECA to block blood flow from the MCA. After 60 min of occlusion, the monofilament was gently withdrawn to recover the blood flow in the MCA (kept under anesthesia). The sham group underwent a similar procedure to the surgical group, but without MCA occlusion. The body temperature of the mice was maintained using a heating pad during the surgery process. All the mice were placed in warm recovery cages after surgery. Upon awakening from the anesthesia, the mice were returned to their cages.

#### 2.2.3. *Scutellaria baicalensis* Extraction Administration

Based on evidence from previous studies, SB was orally administered to an ischemic stroke mouse model at a dose of 200 mg/kg SB [14,16,22]. The vehicle group was administered saline the same number of times as the SB group. The PTB model was treated with saline, SB, or edaravone (Ed; 200 mg/kg) on the day of stroke induction, and the mice were sacrificed the next day. The tMCAO mice were administered SB or saline four times over three days or five times over four days from the day of surgery.

### 2.3. Neurological Severity Score, Weight, and Survival Rate

Neurological deficits were measured using the previous criteria of the Neurological Severity Score (mNSS) on days 1, 3, 7, 14, and 21 after tMCAO modeling [23]. Briefly, the mNSS was used to assess impaired motor, sensory, balance, and reflex function (maximum of 16 points; minimum of 0 points) by ischemic stroke (Table S1). The high mNSS score indicated that severe neurological damage was induced by ischemic stroke. Body weight measurements were also taken up to 21 days after tMCAO modeling. The survival rate was determined over 28 days to evaluate the neuroprotective effects of SB.

### 2.4. 2,3,5-Triphenyl Tetrazolium Chloride Stain

The mice were euthanized by a CO_2_ chamber, and their brains were harvested to assess brain infarct volume using 2,3,5-triphenyl tetrazolium chloride (TTC) staining. The brain was sectioned 2 mm with mouse brain stainless steel matrix (AgnThos, Lidingö, Sweden). The coronally sectioned brains were incubated for 20 min at 37 °C embedding 2% TTC solution and a photograph was taken. The infarct volume was measured using the ImageJ 1.50 software (National Institute of Mental Health, Bethesda, MD, USA) with the obtained images. The infarct volume was calculated for each slice and compared with the contralateral side, excluding the ipsilateral healthy region [24].

### 2.5. Behavior Tests

#### 2.5.1. Open-Field Test 

An open-field test (OFT) was performed to estimate the general locomotor activity after induced ischemic stroke. The tests were assessed in a square open-field arena (40 cm × 40 cm × 40 cm), which was divided into 16 squares, with 4 squares in the center surrounded by 12 peripheral squares. Each mouse was placed in the center of the arena and allowed to explore for 15 min. The experiments were video-recorded, and the total distance was analyzed using EthoVision XT 9.0.

#### 2.5.2. Rotarod Test

Motor function was assessed using the accelerating rotarod test. Before the test, all mice were trained to attain a speed of 8 rpm for 5 min and 12 rpm for 5 min to become accustomed to the rotarod apparatus (B.S. Technolab Inc., Seoul, Republic of Korea). Motor performance was evaluated the day after training at an accelerated speed from 10 to 20 rpm for 10 min. Each mouse was subjected to three trials. The mice were allowed to rest for at least 30 min between each trial to relieve stress and fatigue. The rotarod test was performed for each mouse by averaging the latency times measured in the three trials.

#### 2.5.3. Novel Object Recognition Test

A novel object recognition (NOR) test was conducted to assess changes in cognition and memory in the three groups. The setup consisted of a black-walled square box measuring 40 × 40 × 40 cm. On the first day, the mice were placed in the middle of an open-field box and allowed to adapt for 10 min. The next day, identical objects were placed in the box to perceive each object for 10 min. On the last day, one object was changed to a novel object, and the exploration of the novel or familiar object was recorded for 10 min using the EthoVision XT 9 system (Noldus Information Technology, Wageningen, The Netherlands). The memory index was calculated as the exploration time for each object divided by the total exploration time.

#### 2.5.4. Passive Avoidance Test 

The passive avoidance test (PAT) was performed using a Gemini instrument (San Diego instrument, San Diego, CA, USA, 42.5 cm wide and 35.5 cm long), which was composed of two attached bright and dark boxes. The chambers were divided by a remote operational gate. On the first day, the mice were placed in a light box for 5 min and allowed to freely explore the chambers. The mice were trained to move to a dark box. On the second day, when the mouse was moved to a dark room, the central passage was closed, and an electric shock was applied at 0.2 mA for 5 s. On the third day, the mice were placed in a lit box, and the central passage was opened to measure the time taken to reach the dark box.

### 2.6. Western Blot Analysis

To confirm the protein levels of necroptosis regulators, inflammasomes, before, the brain tissue was stained with 2% TTC solution to isolate the penumbra area (Figure S3). Western blotting was performed as described previously [25]. Briefly, all of the penumbra region was homogenized with radioimmunoprecipitation assay (RIPA) buffer (150 mM NaCl, 1% NP-40, 0.5% sodium deoxycholate, 0.1% SDS, 50 mM Tris, pH 8.0) containing protease inhibitors (Roche Applied Science, Mannheim, Germany) and a cocktail of phosphatase inhibitors (Sigma Aldrich, St. Louis, MO, USA) and centrifuged at 13,000 rpm for 20 min at 4 °C, and the supernatant was collected. The lysate samples were quantified using a bicinchoninic acid assay (Bio-Rad Laboratories, Inc., Hercules, CA, USA). Appropriate amounts of protein were loaded onto an 8–12% sodium dodecyl-sulfate polyacrylamide gel electrophoresis and transferred onto a polyvinylidene difluoride membrane. The membrane was blocked using 3% bovine serum albumin in TBS-T for 1 h at room temperature and then incubated with an appropriate antibody (p-RIPk-1 (Cell Signaling Technology, Danvers, MA, USA, CST #31122s, 1:1000), t-RIPk-1 (Cell Signaling Technology, CST #3493s, 1:1000), p-RIPk-3 (Cell Signaling Technology, CST #91702s, 1:1000), t-RIPk-3 (Santa Cruz Biotechnology, Dallas, TX, USA, sc374639, 1:1000), p-MLKL (Cell Signaling Technology, CST #37333s, 1:1000), t-MLKL (Cell Signaling Technology, CST #37705s, 1:1000), NLRP3 (Novus, Centennial, CO, USA, NBP2-12446, 1:2000), Cleaved caspase-1 (Cell Signaling Technology, CST #89332, 1:1000), ASC (Santa Cruz Biotechnology, sc514414, 1:1000), GSDMD (Santa Cruz Biotechnology, sc393656, 1:2000), Doublecortin (Santa Cruz Biotechnology, sc271390, 1:1000), GAPDH (Santa Cruz Biotechnology, sc32233, 1:10,000)) overnight at 4 °C. After washing three times with TBS-T, the membrane was incubated with a secondary antibody for 1 h. The protein bands were detected using enhanced chemiluminescence (Millipore, Burlington, MA, USA) and BLUE X-ray films (AGFA, Mortsel, Belgium). Band quantification was calculated using the ImageJ software Version 1.4.3.67.

### 2.7. Statistical Analysis

All statistical analyses and outlier removal (significance level: alpha = 0.05) were performed using GraphPad Prism Version 8.2.1 (GraphPad Software Inc., San Diego, CA, USA). All graph data are presented as the mean ± the standard error (SEM). The data collected from the NOR memory index and mNSS test between the groups were analyzed using an unpaired *t*-test. Body weight was analyzed using a two-way analysis of variance (ANOVA) followed by Tukey’s multiple comparison test. The behavioral test and TTC staining data were analyzed using a one-way ANOVA, followed by Tukey’s multiple comparison test. Differences in the survival rates between the groups were analyzed using the log-rank (Mantel–Cox) test. A *p*-value of <0.05 was considered statistically significant (* *p* < 0.05; ** *p* < 0.01; *** *p* < 0.001; and **** *p* < 0.0001).

## 3. Results

### 3.1. SB Improved Neurological Function in the Mouse Model of PTB Stroke

**Figure 1:**
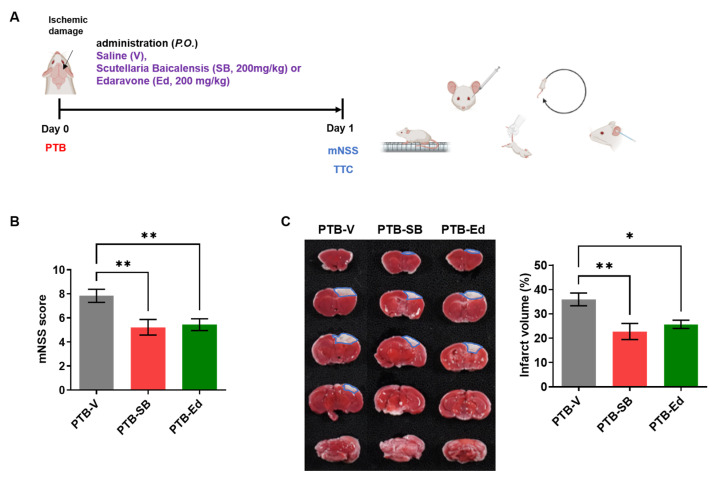
The therapeutic effects of SB were confirmed in the PTB model compared with Edaravone. (**A**) Experiment schedule design with the PTB mouse model. (**B**) The neurological severity score (mNSS) was measured before sacrificing the PTB model mice (PTB-V, *n* = 48; PTB-SB, *n* = 28; PTB-Ed, *n* = 39). (**C**) The 2,3,5-triphenyl tetrazolium chloride stain represents the infarct region in the PTB model one day after modeling. The infarct area is denoted by a blue line. Quantification of each group’s infarct region volume (PTB-V, *n* = 8; PTB-SB, *n* = 9; PTB-Ed, *n* = 14). Mean ± SEM, * *p* < 0.05, ** *p* < 0.01, one-way analysis of variance followed by Turkey’s multiple comparison tests.

**Figure 2:**
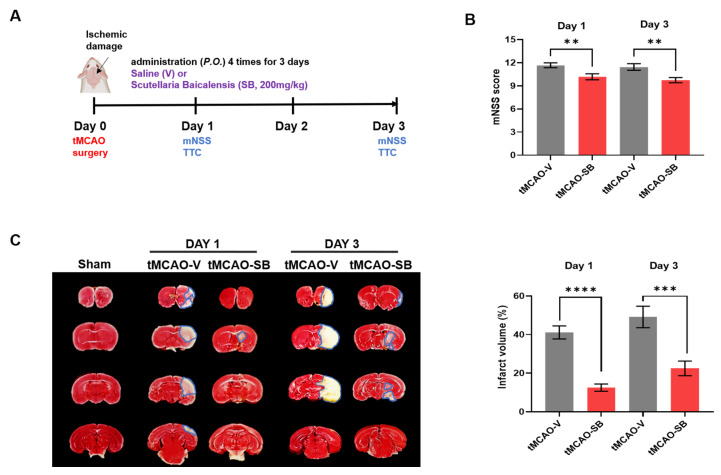
SB administration attenuated ischemic damage in the tMCAO stroke mice model. (**A**) Experiment schedule of the evaluation of the therapeutic impact of SB in the acute ischemic stroke phase of the tMCAO mice model. (**B**) The mNSS was assessed one and three days after tMCAO modeling (Day 1: tMCAO-V, *n* = 21; tMCAO-SB, *n* = 22, Day 3: tMCAO-V, *n* = 20; tMCAO-SB, *n* = 22). Mean ± SEM, ** *p* < 0.01, one-tailed *t*-test. SB was administrated four times after tMCAO modeling until Day 3. (**C**) The 2,3,5-triphenyl tetrazolium chloride staining results on Days 1 and 3 for each group. The infarct region is shown by a blue line. Quantification of the infarct region volume compared with the contralateral side (tMCAO-V, *n* = 6; tMCAO-SB, *n* = 6). Mean ± SEM, *** *p* < 0.001, **** *p* < 0.0001 vs. tMCAO-V. Statistical analyses were performed using a one-way analysis of variance followed by Tukey’s multiple comparison test.

Since the acute stage of stroke is the most important in terms of mortality and sequelae, the protective effects of SB counter to ischemic brain injury in the PTB stroke and tMCAO mouse models were investigated (Figure 1 and Figure 2). The effects of SB were preliminarily tested in the mouse model of PTB stroke (Figure 1A), which was generated by inducing cerebral infarction through the photoactivation of a light-sensitive dye. Focal thrombosis-induced ischemic lesions appeared in the sensorimotor cortices of the ICR mice 24 h after photoirradiation. After establishing the PTB stroke model, SB was administered at a dose of 200 mg/kg [22], along with Ed as the positive control. Ed was previously found to have therapeutic effect in the mouse model of tMCAO stroke [26]. Neurological deficits were assessed using the mNSS, which assesses the stroke severity in terms of body balance, limb force, reflection, and walking ability (Figure 1A). The day after modeling, each stroke mouse model was evaluated using the mNSS, and the mNSS of the SB- and Ed-treated PTB mice was significantly reduced compared to the vehicle (V)-treated PTB mice (PTB-V, 8 ± 1; PTB-SB, 5 ± 1, ** *p* < 0.01; PTB-Ed, 5 ± 1, ** *p* < 0.01) (Figure 1B). To determine the protective effects of SB against acute brain injury in PTB mice, the mouse brains were extracted, and the brain infarction was quantified using TTC staining. The infarct volume of the PTB-SB group was significantly decreased compared to that of the PTB-V group (PTB-V, 35.9 ± 7.8 mm^3^; PTB-SB, 22.7 ± 9.3 mm^3^, ** *p* < 0.01; PTB-Ed, 25.7± 9.3, * *p* < 0.05) (Figure 1C). 

Next, we confirmed the protective effects of SB counter to acute ischemic injury in mice through tMCAO and reperfusion by quantifying the brain infarction and assessing the mNSS on Days 1 and 3 after tMCAO (Figure 2A). The mNSS in the group of tMCAO mice applied with SB (tMCAO-SB) was meaningfully decreased compared to the group of tMCAO mice treated with the vehicle (tMCAO-V) on Days 1 (tMCAO-V, 11 ± 1; tMCAO-SB, 10 ± 1, ** *p* < 0.01) and 3 (tMCAO-V, 11 ± 1; tMCAO-SB, 9 ± 1, ** *p* < 0.01) after stroke onset (Figure 2B). TTC staining showed the infarct volume (%) in the brains of the tMCAO mice on Days 1 and 3 after tMCAO modeling compared to the sham (surgery control) mice (Figure 2C). On Day 1 after the stroke onset, the tMCAO-SB group had a significantly reduced infarct volume compared to the tMCAO-V group (tMCAO-V, 41.6 ± 5%; tMCAO-SB, 12.5 ± 7%, **** *p* < 0.0001) (Figure 2C). On Day 3 after the stroke onset, the infarction volume of the tMCAO mice was more increased than on Day 1, and SB treatment significantly reduced the infarction volume compared with the tMCAO-V group (tMCAO-V, 49.1 ± 9%; tMCAO-SB, 22.5 ± 7%, *** *p* < 0.001) (Figure 2C). These data indicate that SB protects neurons from ischemic damage during the acute stage of stroke.

### 3.2. SB Downregulated the Key Regulators of Necroptosis in tMCAO Mice Brains

**Figure 3:**
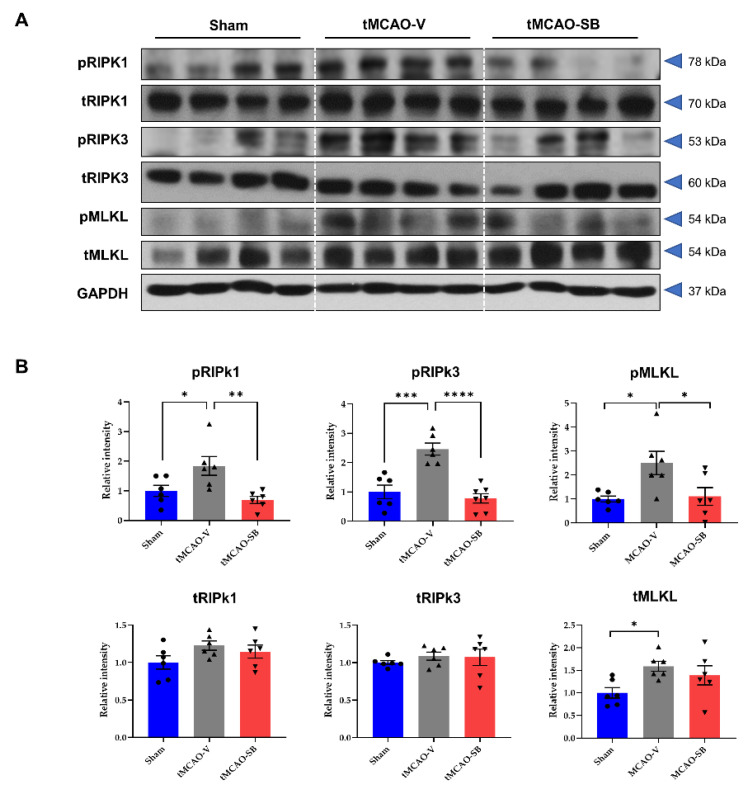
SB decreased necroptosis regulator proteins in tMCAO mice brains. Western blot analysis was performed on the ipsilateral side penumbra region of each mouse brain 3 days after tMCAO. (**A**) Representative Western blots of pRIPK1, tRIPK1, pRIPK3, tRIPK3, pMLKL, tMLKL, and GAPDH. (**B**) The ratio of the phosphorylated form to the total forms of RIPK1, RIPK3, and MLKL was quantified (sham, *n* = 6; tMCAO-V, *n* = 6; tMCAO-SB, *n* = 6). Mean ± SEM, * *p* < 0.05, ** *p* < 0.01, *** *p* < 0.001, **** *p* < 0.0001. Statistical analyses were performed using a one-way analysis of variance followed by Tukey’s multiple comparison test.

Although necroptosis is a crucial programmed cell death pathway that is closely associated with myocardial ischemia/reperfusion injury [27], whether SB modulates necroptosis in an ischemic stroke model remains to be elucidated. RIPK1 and RIPK-3 assemble to form the RIPK1/RIPK3 necrosome, and MLKL is a substrate for RIP3 kinase. It has been previously reported that necroptosis regulators (RIPK1, RIPK3, and MLKL) increase significantly on Day 3 after ischemia/reperfusion injury [28]. Therefore, we determined the protein levels of the necroptosis regulators, RIPK1, RIPK3, and MLKL, to elucidate the influence of SB on the mechanism of necroptosis. On Day 3 after tMCAO, the protein levels of the phosphorylated forms and total forms of RIPK1, RIPK3, and MLKL were detected in the penumbra of the brains of the sham, tMCAO-V, and tMCAO-SB groups (Figure 3A). The protein levels of the phosphorylated forms of RIPK1, RIPK3, and MLKL (pRIPK1, pRIPK3, and pMLKL) were significantly increased in the tMCAO-V group compared to the sham group (pRIPK1, 1.842 ± 0.317, * *p* < 0.05; pRIPK3, 2.463 ± 0.287, *** *p* < 0.001; pMLKL; 2.503 ± 0.508, * *p* < 0.05), but decreased in the tMCAO-SB group compared to the tMCAO-V group (pRIPK1, 0.693 ± 0.317, ** *p* < 0.01; pRIPK3, 0.781 ± 0.276, **** *p* < 0.0001; pMLKL; 1.108 ± 0.508, * *p* < 0.05). There were no significant differences from sham and tMCAO-SB groups (Figure 3B). 

The total protein levels of MLKL (tMLKL) in the tMCAO-V group were significantly higher than in the sham group (tMLKL; 1.590 ± 0.217, * *p* < 0.05), but there was no difference between the tMCAO-SB group and the tMCAO-V group (Figure 3B). However, the total protein levels of RIPK1 and RIPK3 (tRIPK1 and tRIPK3) did not differ between the sham groups, tMCAO-V, and tMCAO-SB (Figure 3B). These findings show that SB downregulates the kinase activities of RIPK1, RIPK3, and MLKL during the acute stage of stroke.

### 3.3. SB Downregulated NLRP3 Inflammasome and the Key Regulators of Pyroptosis in tMCAO Mice Brains

**Figure 4:**
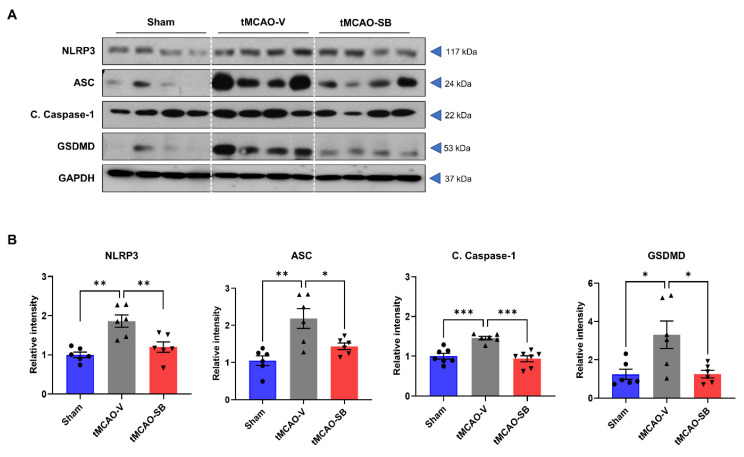
SB decreased the pyroptosis regulator proteins in tMCAO mice brains. Western blot analysis was performed on the ipsilateral side penumbra region of each mouse brain 3 days after tMCAO. (**A**) Representative Western blots of NLRP3, ASC, C. caspase-1, GSDMD, and GAPDH. (**B**) Protein levels of the pyroptosis regulatory factors (NLRP3, ASC, C. caspase-1, GSDMD) were detected by Western blotting on Day 3 in the penumbra region of each brain. Protein levels were quantified against those of GAPDH (sham, *n* = 6; MCAO-V, *n* = 6; MCAO-SB, *n* = 6). Mean ± SEM, * *p* < 0.05, ** *p* < 0.01, *** *p* < 0.001. Statistical analyses were performed using a one-way analysis of variance followed by Tukey’s multiple comparison test.

Pyroptosis, a form of programmed cell death initiated by the generation of the NLRP3 inflammasome, which promotes caspase 1 activation, is involved in the initiation and development of ischemic stroke. Upon cleavage, caspase 1 promotes gasdermin D (GSDMD) pore formation, resulting in cell death and an immunogenic response [29]. To investigate the changes in the modulators of inflammasomes and pyroptosis following treatment with SB, we determined the protein levels of NLRP3, ASC, cleaved caspase-1 (C. caspase-1), and GSDMD in the penumbra area of the brain (Figure 4A). On Day 3 of tMCAO, the protein level of NLRP3 was significantly increased in the tMCAO-V group compared to the sham group (tMCAO-V, 1.86 ± 0.16, ** *p* < 0.01) and decreased in the tMCAO-SB group compared with the tMCAO-V group (tMCAO-SB, 1.2 ± 0.13, ** *p* < 0.01) (Figure 4B). The protein levels of ASC and C. caspase-1 were also higher in the tMCAO-V group than in the sham group (tMCAO-V: ASC, 2.18 ± 0.27, ** *p* < 0.01; C. caspase-1, 1.46 ± 0.04, *** *p* < 0.001) and lower in the tMCAO-SB group than in the tMCAO-V group (tMCAO-SB: ASC, 1.43 ± 0.09, * *p* < 0.05; C. caspase-1, 0.93 ± 0.07, *** *p* < 0.001) (Figure 4B). The tMCAO-V group had a higher level of GSDMD than the sham group (3.30 ± 0.72, * *p* < 0.01) and the tMCAO-SB group (1.26 ± 0.19, * *p* < 0.05). There was no meaningful differentiation of the sham and tMCAO-SB group (Figure 4B). These results suggest that SB downregulates the key molecules involved in pyroptosis during the acute stage of stroke.

### 3.4. SB Administration Showed Long-Term Neuroprotective Effects against Brain Injuries following tMCAO

On the third and tenth days after modeling, micro positron emission tomography (PET) with [^18^F] fluorodeoxyglucose (FDG) was used to confirm whether SB affected metabolic activity. Glucose uptake was lower in the ipsilateral region than in the contralateral region in the tMCAO-V group and increased more in the tMCAO-SB group than in the tMCAO-V group (tMCAO-V, 70 ± 11%, tMCAO-SB, 93 ± 16%, * *p* < 0.05) on Day 3 (Figure S4A). We also checked whether SB affects metabolic activity one week after SB treatment and found that glucose uptake was still increased more in the tMCAO-SB group than in the tMCAO-V group (tMCAO-V, 83 ± 13%, tMCAO-SB, 101 ± 12%, * *p <* 0.05) on Days 10 to 11 (Figure S4B,C). These results show that the effects of SB on neuroprotection and metabolic activity were observed even after SB treatment in the tMCAO mouse model.

**Figure 5:**
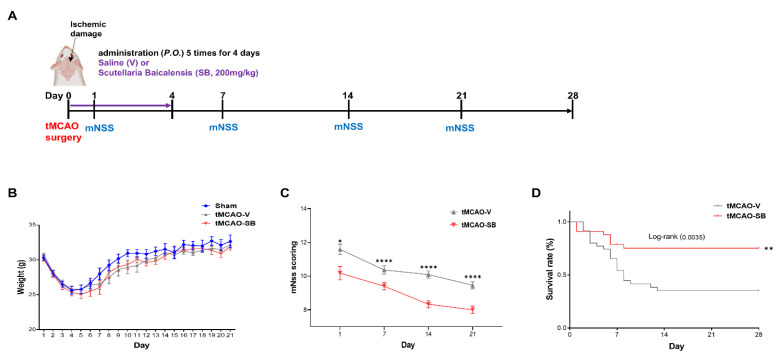
SB treatment in the acute stroke phase improves the survival rate and neurological function. (**A**) Schedule of the mNSS conducted on the tMCAO mouse model over 4 weeks. The mNSS was measured on Days 1, 7, 14, and 21. (**B**) The weight of each mouse was measured daily for 28 days. (**C**) mNSS (tMCAO-V, *n* = 22; tMCAO-SB, *n* = 23). Statistical analysis was performed on Days 1, 7, 14, and 21 for each group using an unpaired one-tail *t*-test. Mean ± SEM, * *p* < 0.05, ** *p* < 0.01, **** *p* < 0.0001 vs. tMCAO-V. (**D**) Survival rate (tMCAO-V, *n* = 35; tMCAO-SB, *n* = 33). Log-rank match SPSS and SAS 95% CI asymmetrical were performed in each group.

Next, we investigated whether the neuroprotective effects of SB on ischemic brain impairment after tMCAO could be preserved to the 28 days in the tMCAO mouse model administered the vehicle or SB for four days at the onset of stroke (Figure 5A and Figure S5). We continuously monitored the weights of all mice for 21 days and found no notable differences between the groups (Figure 5B). The mNSS was assessed on Days 1, 7, 14, and 21 after tMCAO surgery, which found that SB application mitigated neurological deficits more in the tMCAO-SB group (10.17 ± 0.39; 9.40 ± 0.21; 8.33 ± 0.21; and 8.00 ± 0.22; ** *p <* 0.05, **** *p <* 0.0001) than in the tMCAO-V group (11.59 ± 0.31; 10.365 ± 0.24; 10.09 ± 0.21; and 9.46 ± 0.21) at 1, 7, 14, and 21 days after tMCAO induction (Figure 5C). The survival rate of the tMCAO-SB group was significantly higher than that of the tMCAO-V group at 28 days (** *p* < 0.01) (Figure 5D).

**Figure 6:**
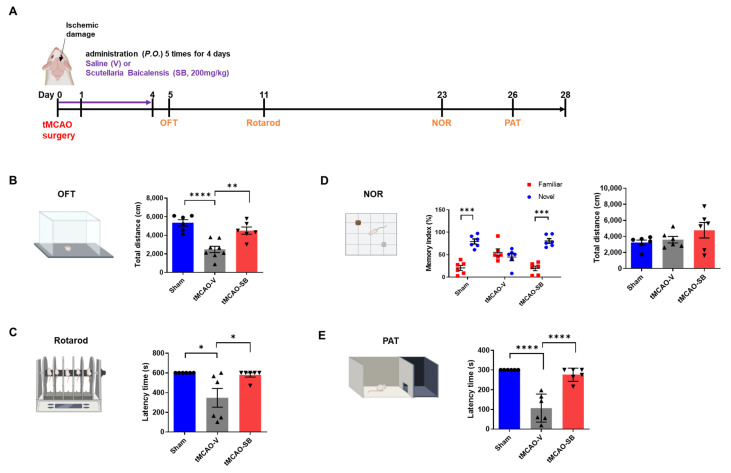
SB treatment in the acute stroke phase recovers the sequela in motor and cognitive impairment by ischemic stroke. (**A**) Schedule of the behavioral tests conducted on the tMCAO model mice over 4 weeks. (**B**) The open-field test (OFT) was performed five days after tMCAO modeling (sham, *n* = 6; tMCAO-V, *n* = 8; tMCAO-SB, *n* = 6). (**C**) The rotarod test was performed 11 days after tMCAO modeling (sham, *n* = 6; tMCAO-V, *n* = 6; tMCAO-SB, *n* = 6). (**D**) The novel object recognition (NOR) test was performed at 23 days. The OFT was performed five days after tMCAO modeling. The memory index was calculated as the difference in time as a percentage of the total time exploring the two objects. Statistical analyses were performed using a two-way analysis of variance (ANOVA) followed by Bonferroni’s multiple comparisons test. (**E**) The passive avoidance test (PAT) was performed 26 days after tMCAO modeling (sham, *n* = 6; tMCAO-V, *n* = 6; tMCAO-SB, *n* = 6). Mean ± SEM * *p* < 0.05, ** *p* < 0.01, *** *p* < 0.001, **** *p* < 0.0001). Statistical analyses were performed using a one-way ANOVA followed by Tukey’s multiple comparison test.

To evaluate motor function after tMCAO modeling, the OFT was performed on Day 5, and the rotarod test was performed on Day 11 (Figure 6A). We hypothesized that the tMCAO-SB group would increase the total distance in the OFT compared to the tMCAO-V group. In the OFT, the total travel distance was calculated for each group to estimate the motor ability after inducing ischemic damage. The tMCAO-V group was lower than the sham group (sham, 5335 ± 347.3; tMCAO-V, 2478 ± 341.7, **** *p <* 0.0001), and that of the tMCAO-SB group was higher than that of the tMCAO-V group (tMCAO-SB, 4504 ± 388.5, ** *p <* 0.01). There was no significant difference between the sham and tMCAO-SB groups (Figure 6B). We anticipated the tMCAO-SB group had a longer latency than tMCAO-V in the rotarod test. In the rotarod test, the tMCAO-SB group showed a longer latency than the tMCAO-V group, which had a lower latency than the sham group (sham, 600 ± 0; tMCAO-V, 374.9 ± 94.98, * *p <* 0.05; tMCAO-SB, 578.6 ± 21.44, * *p <* 0.05) (Figure 6C). This indicates that the motor function of the MCAO-SB group was improved compared to that of the tMCAO-V group. There was no difference between the sham and tMCAO-SB group.

To identify the effect of SB on cognitive impairment after stroke, the NOR test and PAT were performed on Days 23 and 26, respectively (Figure 6A). The NOR test assesses recognition memory, while the PAT is utilized to identify short-term memory capabilities. In the NOR test, the memory index, which indicates the exploratory rate, was significantly increased for a novel object compared with familiar objects in the sham and tMCAO-SB groups (sham, 20.52 ± 5.63 vs. 79.48 ± 5.63, *** *p <* 0.001; tMCAO-SB, 19.43 ± 5.19 vs. 80.57 ± 5.19, *** *p <* 0.001). However, the memory index of the tMCAO-V group did not differ between the novel and familiar objects (tMCAO-V; 55.22 ± 7.89 vs. 44.78 ± 7.89, ns) (Figure 6D). There were no significant differences in the total distance explored within each group (sham: 3221 ± 317.3; tMCAO-V, 3592 ± 404.7; tMCAO-SB, 4771 ± 961.5) (Figure 6D). In the PAT, which assesses short-term memory, the tMCAO-SB group showed a high latency time compared to the tMCAO-V group, which had a lower latency time than the sham group (sham; 300 ± 0, **** *p <* 0.0001; tMCAO-V, 106.8 ± 29.12; tMCAO-SB, 276 ± 13.48, **** *p <* 0.0001) (Figure 6E). These results indicate that ischemic stroke causes motor and cognitive impairments and that SB administration improved these deficits in the tMCAO mice.

## 4. Discussion

Stroke can result in persistent disabilities even after recovery and is associated with a significant socioeconomic burden. In the United States, stroke has been estimated to affect 800,000 individuals annually, resulting in approximately 140,000 stroke-related fatalities [30,31]. Notably, the incidence of ischemic stroke in young adults has exhibited an alarming upward trend, which is attributed to lifestyle-related factors. Stroke is a life-threatening and costly pathological condition that requires substantial financial investment for patient management. Currently, tissue plasminogen activator (TPA) is the only Food and Drug Administration-approved pharmacological intervention for stroke. However, the therapeutic scope of TPA is limited to clot dissolution, rendering it devoid of substantive clinical benefits. Its administration window is restricted to a narrow 4.5 h timeframe following stroke onset [32,33,34]. Moreover, TPA exerts adverse effects, including disruption of the BBB’s integrity and activation of matrix metalloproteinases 2 and 3, predisposing patients to cerebral hemorrhage [34]. Consequently, there is a pressing need to develop novel pharmaceutical agents that target stroke pathology.

Recent studies have focused on investigating the role of the immune system in stroke pathogenesis, emphasizing the involvement of inflammatory responses, immune cell infiltration, compromised BBB integrity, and various cell death mechanisms, including apoptosis, necroptosis, and pyroptosis [35,36,37,38,39]. Necroptosis, characterized by the phosphorylation of RIPK1 and RIPK3, leads to MLKL phosphorylation and the subsequent formation of necrosomes, inducing nanoscale pores in the cell membrane [40]. In addition, pyroptosis, which is characterized by rapid plasma membrane rupture, involves GSDMD activation via caspase activation during inflammasome formation [41,42].

The extract of *Scutellaria baicalensis* has been studied by being applied to various diseases in Oriental medicine or Western medicine while showing various effects such as anticancer, antioxidant, and anti-inflammatory [43,44]. It has also been applied to research on ischemic stroke, and it has been confirmed that oxidative stress caused by ischemic stroke, the breakdown of mitochondria, and abnormalities in cellular metabolic processes induce changes at the gene level [45]. In addition, as evidenced from recent studies, SB has been found to affect anti-inflammation, nerve regeneration, and the permeability of the blood–brain barrier by stroke [46], and it has been reported to affect ferroptosis, a new cell death process [17,22,47,48]. In addition, we conducted acute and chronic toxicity evaluations of a mixture containing SB in the following papers and found no toxicity issues with SB [49]. Furthermore, the traditional usage of SB listed in textbooks is more than 4 g per day. This is similar to the dose described in representative traditional literature such as *Donguibogam*, *Ben Cao Gang Mu*, *Zhong Hua Ben Cao*, etc. Based on this, we calculated the safe dose for mouse experiments based on the correlation between animal and human equivalent doses and extraction yield, and as a result, it came out to about 200 mg/kg for the mouse [50].

Our findings showed the neuroprotective effects of SB against ischemic brain damage in two mouse models: PTB-induced permanent ischemia and surgically induced tMCAO, which mimic human ischemic stroke by inducing large infarcts in multiple regions of the ipsilateral brain [51]. The PTB-induced model specifically targets superficial vessels without neovascularization or observable reperfusion damage [19,52,53,54]. Previous studies have primarily focused on the anti-apoptotic properties of SB during ischemic injury [55,56]. However, the regulatory effects of SB on necroptosis and pyroptosis during ischemic stroke remain largely unknown. Thus, we aimed to elucidate the ability of SB to modulate the key regulators of necroptosis (RIPk-1, RIPk-3, and MLKL) and pyroptosis-inducing inflammasome complexes (NLRP3, ASC, C. caspase-1, GSDMD).

Recently, the concept of PANoptosis, a regulated cell death pathway observed in ischemia/reperfusion models, has emerged [57]. PANoptosis is governed by the PANoptosome complex, which exhibits the key characteristics of pyroptosis, apoptosis, and necroptosis [58]. This complex comprises three classes of proteins: (1) ZBP1 and NLRP3, which serve as putative sensors for damage-associated molecular patterns; (2) ASC and FADD, which function as adaptors; and (3) RIPK1, RIPK3, caspase-1, and caspase-8, which act as catalytic effectors [59]. The discovery of PANoptosis in the context of ischemic stroke may lead to new avenues for stroke treatment and provide valuable insights for stroke research. Targeting PANoptosis has been proposed as a novel approach to stroke management. Furthermore, it has been hypothesized that SB may modulate the PANoptotic pathway during ischemic stroke. By investigating the regulation of PANoptosis, SB holds promise as a therapeutic intervention for the treatment of ischemic stroke.

Cerebral ischemia, characterized by a restricted blood supply to the brain, leads to impaired sensory and motor abilities and neuronal injury. The accurate assessment of functional outcomes is crucial because many patients with stroke experience post-stroke dysfunction. The acute phase of stroke is a pivotal stage that involves immune response and neuronal dysfunction and determines survival. Post-stroke cognitive impairment, a prevalent complication, often remains undetected because of overshadowing symptoms, such as motor or visual disturbances. In this study, we used the tMCAO model to evaluate motor and cognitive functions. Our results demonstrated that treatment with SB in the early stages of tMCAO significantly ameliorated motor and cognitive function compared to the tMCAO-V group, as assessed through behavioral tests. Notably, these improvements were observed after only five SB treatments, highlighting the importance of early stroke interventions. Furthermore, the quantification of brain tissue loss (%) revealed a more-pronounced reduction in the tMCAO-SB group than in the tMCAO-V group after four weeks. This indicates that SB treatment exerts neuroprotective effects by limiting the extent of brain tissue damage. In support of this, our study found sustained anti-inflammatory effects of SB, as evidenced by reduced levels of NLRP3 (a key inflammatory protein) and active C. caspase-1 (an enzyme involved in the inflammatory response) in the chronic tMCAO model treated with SB compared to the tMCAO-V group. Collectively, these findings underscore the therapeutic potential of early SB intervention in patients with stroke. Moreover, they highlight the significance of SB in mitigating long-term functional disabilities by attenuating neuronal damage, preserving the brain tissue, and modulating inflammatory processes.

SB contains hundreds of components, including flavonoids, volatile oils, terpenoids, polysaccharides, phenylethyls, amino acids, sterols, starches, alkaloids, organic acids, and trace elements. Flavonoids are the main components of SB. There are more than 100 flavonoids in SB, and certain 4′-deoxypavones such as chrysin, baicalein, wogonin, and their glycosides (baicalin and wagonosides) are the main pharmacologically active components [18]. We will study the synergistic effects of extracts, and their detailed modulating pathway is something that needs to be further explored.

In conclusion, our study provides strong evidence for the neuroprotective effects of SB treatment in the acute stage of stroke. These effects are achieved by modulating PANoptosis and reducing inflammation, leading to improvements in the long-term motor and memory dysfunction observed in stroke sequelae. Based on these findings, we propose SB as a potential therapeutic drug candidate for stroke treatment. Further research and clinical trials are needed to fully explore its efficacy and safety in patients with stroke.

## Data Availability

The data supporting the findings of this study are available from the corresponding author upon reasonable request.

## Supplementary Materials

The following supporting information can be downloaded at: https://www.mdpi.com/article/10.3390/cells12172133/s1, Supplementary method: MicroPET imaging using 18[F]-FDG; Table S1: Modified neurological severity score criteria; Figure S1: The compounds in Scutellaria baicalensis (SB) analyzed by high-performance liquid chromatography; Figure S2: Representative data of blood flow tracked by laser Doppler blood flowmeter; Figure S3: Indicated each region in brain tissue after ischemic stroke induced; Figure S4: 18[F]-FDG uptake in the brains of the tMCAO model mice scanned by microPET on days 3 and 10 to 11; Figure S5: The schedule of long-lasting ischemic stroke model.
