# Supplementary material for: Scutellaria baicalensis Attenuated Neurological Impairment by Regulating Programmed Cell Death Pathway in Ischemic Stroke Mice
Source: Cells. 2023 Aug 23;12(17):2133. doi: 10.3390/cells12172133 (PMC10486384; doi:10.3390/cells12172133)
Supplement: Supplementary file 1 [file cells-12-02133-s001.zip › cells-2543700-supplementary.pdf]

Supplementary Method

MicroPET imaging using ^{18}[F]-FDG

MicroPET experiments and analysis were done using the microPET scanner (Focus 120 MicroPET, Concorde Microsystems, Knoxville, TN, United States) at Core-facility for Cell to In-vivo imaging to create the maps indicating glucose metabolic activity using tMCAO mice with SB or Vehicle at the time of 3 days. Prior to microPET scanning, mice were kept in cages for 50 min in a room maintained at 30◦C to maximize 18F-FDG uptake, as previously described (Fueger et al., 2006). For the acquisition of PET signal, approximately 250 μCi of 18F-FDG was injected via tail vein under the light isoflurane anesthesia. Mice then remained in an awake state in the dark cage for another 30 min to induce the 18F-FDG uptake. The PET images were acquired 50 min after intravenous injection in axial mode for 20 minutes. During the PET scan, mice were anesthetized under 2% isoflurane.

Supplementary Table

Test	Score
**Beam balance test**	**Maximum = 6**
● Keep/not the body balance on the beam.	0 / 1
● Forelimbs hug side of beam	1
● Hindlimbs hug side of beam	1
● Unable to walk on top of beam, but get back up the body balance.	1
● Unable the get back up the body balance but fall off (>40 seconds)	1
● Unable to hang on to beam	1
**Placing the mouse on the floor**	**Maximum = 3**
● Walking straight / Walking un-straight	0/1
● Circling	2
● The body fall down one side	3
**Raising mouse by the tail**	**Maximum = 3**
● Forelimbs flex abnormality	1
● Hindlimbs flex abnormality	1
● Head shaking (>10°)	1
**Reflex absence and abnormal movements**	**Maximum = 4**
● Pinna reflex	1
● Corneal reflex	1
● Startle reflex	1
● Seizure and grip strength of forelimbs and hindlimbs	1
**Total**	**16**

Table S1. Modified neurological severity score criteria It was largely experimented with 5 types of tests (Beam balance, Placing the mouse on the floor, Raising mouse by the tail, Sensory and Reflex absence, and abnormal movements). The final score is 16, with abnormal mice representing higher scores.

Supplementary Figures

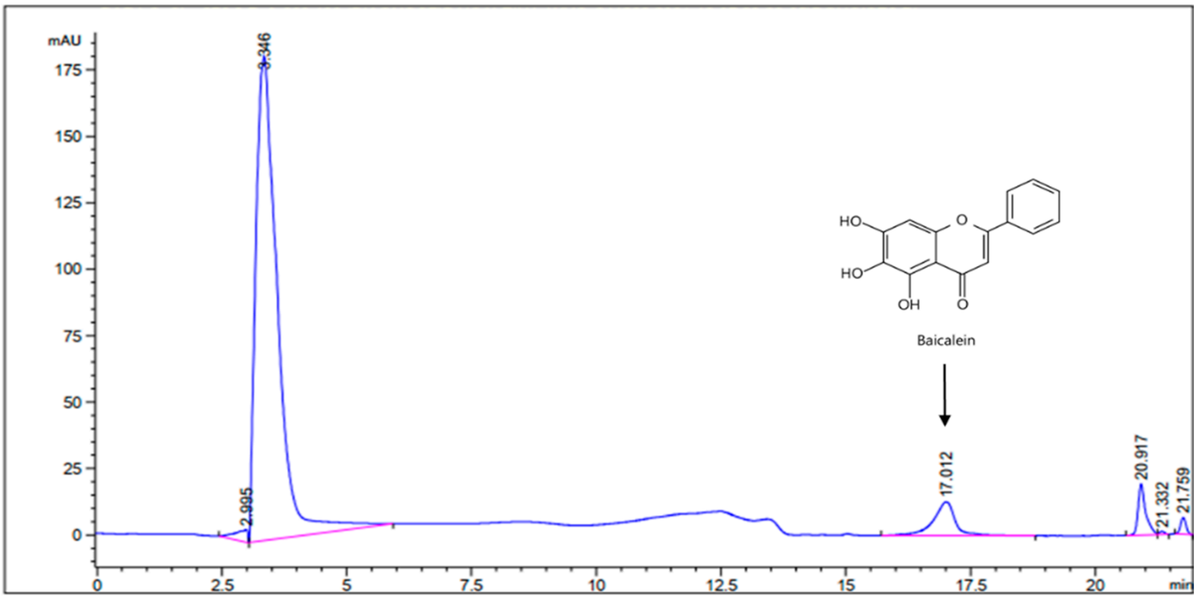

Figure S1. The compounds in *Scutellaria baicalensis* (SB) analyzed by high-performance liquid chromatography. Chromatographic analysis of SBE was performed by high-performance liquid chromatography (HPLC) using a 1100 series HPLC system (Agilent, USA). A Zorbax Eclipse XDB C18 column (4.6 × 250 mm, 5 μm; Agilent) was used for chromatic separation at 30 °C. A 1 mg sample was diluted with 10 mL of 50% methanol and then sonicated for 10 min. Samples were filtered using a 0.45 μm syringe filter (Waters Corp., USA). The mobile phase components were 0.1% formic acid and acetonitrile with the column flowed out as follows: 0–3 min, 20%; 3–15 min, 20%–45%; 15–20 min, 45%–60%; and 20–22 min, 60% solvent B. A 10 μL injection volume was used to mark the runoff at 276 nm. The analysis was performed three times. Baicalein was identified in the SB using the HPLC-UV method.

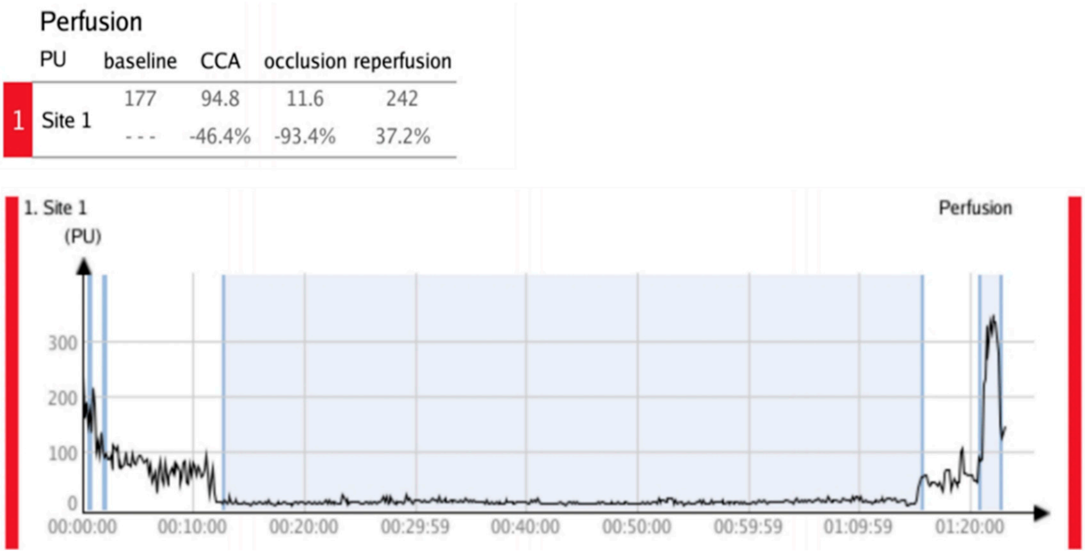

Perfusion

PU		baseline	CCA	occlusion	reperfusion
1	Site 1	177	94.8	11.6	242
		- - -	-46.4%	-93.4%	37.2%

Figure S2. Representative data of blood flow tracked by laser Doppler blood flowmeter

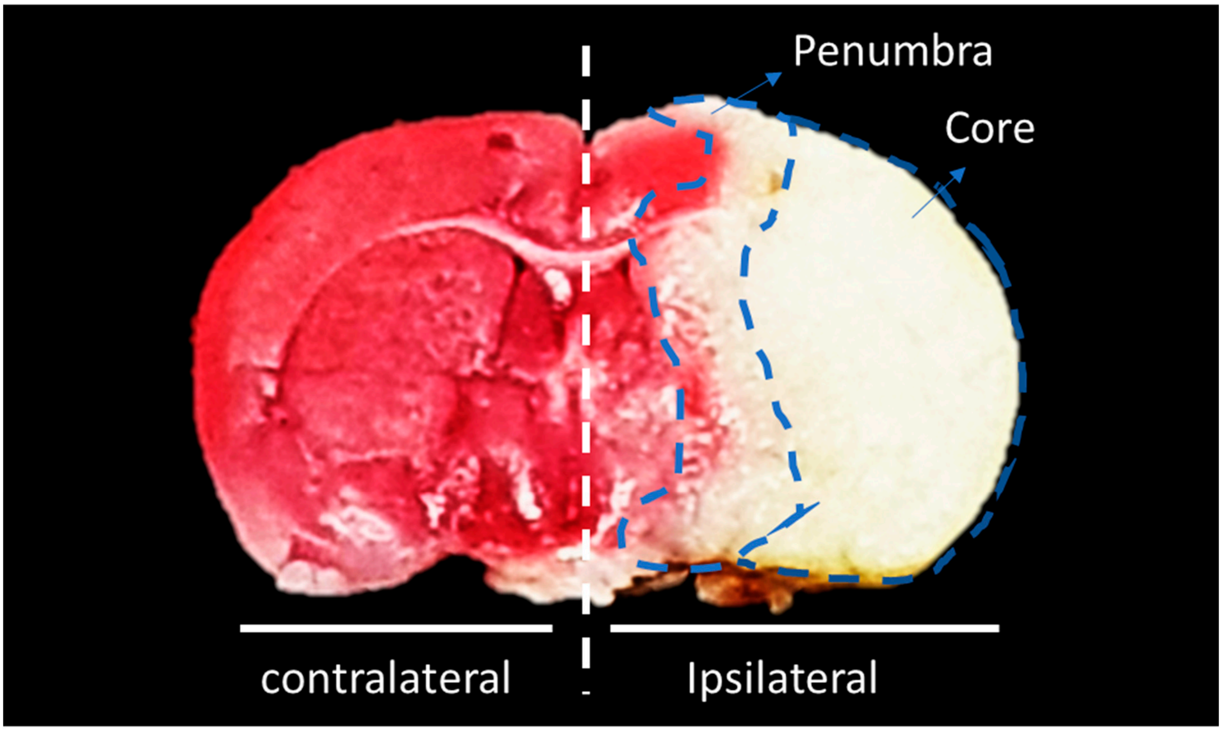

Figure S3. Indicated each region in brain tissue after ischemic stroke induced.

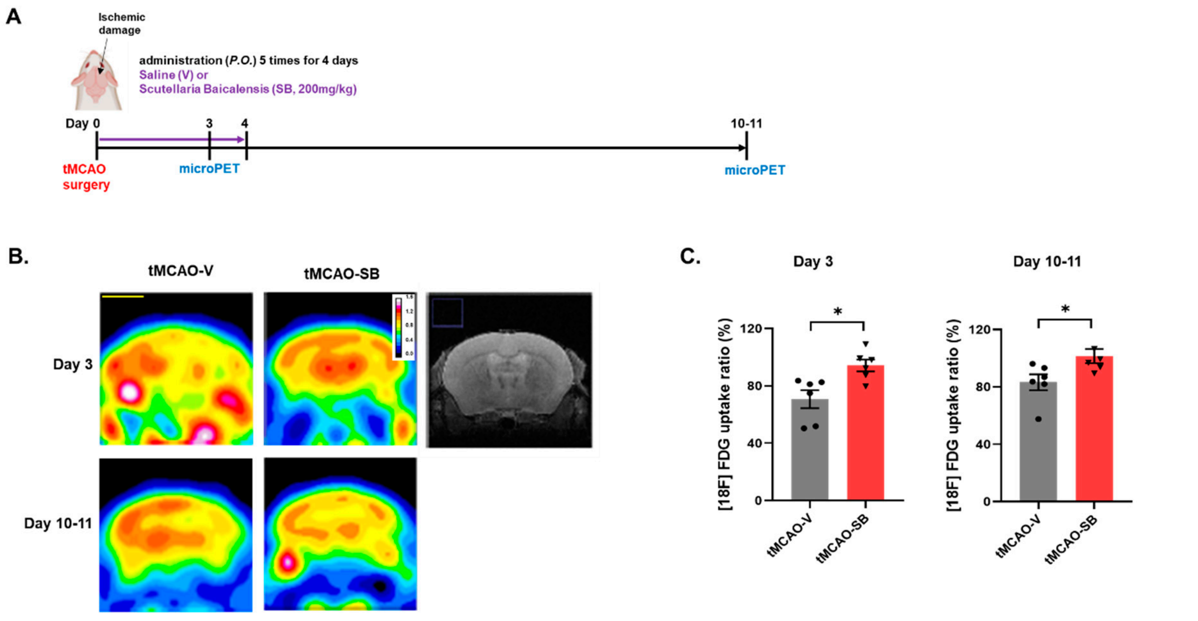

Figure S4. [18][F]-FDG uptake in the brains of the tMCAO model mice scanned by microPET on days 3 and 10 to 11. (A) The schedule of microPET imaging with tMCAO mouse model. Each group was randomly divided into tMCAO-V and tMCAO-SB. tMCAO mice were orally administrated with V or SB once per day for 4 days. (B) representative [18] [F]-PET scan image each group in day 3 and 10-11 from tMCAO modeling. (C) Quantified data against the relative SUV (tMCAO-V, n = 6; tMCAO-SB, n = 7). Mean ± SEM, $^{**}p < 0.01$, one-tailed t-test.

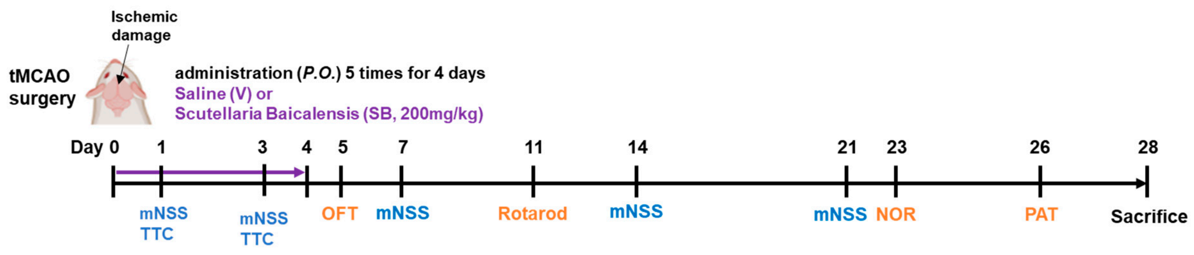

Figure S5. The schedule of long-lasting ischemic stroke model
